# Intensive care nurses’ knowledge and practice on endotracheal suctioning of the intubated patient: A quantitative cross-sectional observational study

**DOI:** 10.1371/journal.pone.0201743

**Published:** 2018-08-16

**Authors:** Emelia T. Mwakanyanga, Golden M. Masika, Edith A. M. Tarimo

**Affiliations:** 1 MUHAS Academic Medical Centre (MAMC), Dar es Salaam, Tanzania; 2 School of Nursing, College of Health Sciences, The University of Dodoma, Dodoma, Tanzania; 3 Department of Nursing Management, School of Nursing, Muhimbili University of Health and Allied Sciences, Dar es salaam, Tanzania; University of Central Florida, UNITED STATES

## Abstract

**Introduction:**

Endotracheal suction (ETS) is a common invasive procedure which is done to keep the airways patent by mechanically removing accumulated pulmonary secretions to all in patients with artificial airways. Many life-threatening complications can occur when the procedure is not performed correctly. Although the evidence-based recommendations regarding ETS are available, many of these have not been observed in nurse’s clinical practice. We assessed the intensive care nurses’ knowledge and practice of ETS to intubated patients in selected hospitals in Dar es Salaam, Tanzania.

**Methodology:**

A descriptive cross-sectional study design involving 103 Intensive Care Unit (ICU) nurses in Dar es Salaam city was conducted in 2014. Data were analyzed using SPSS version 20 where descriptive statistics were employed to interpret data.

**Results:**

Majority of ICU nurses (69.9%) knew the indication for the procedure, (77.7%) knew the action to be taken in case of abrupt change in the ECG monitor; however, 80.6% demonstrated undesirable overall knowledge on ETS evidence-based recommendations. Nurses with ICU training (57.3%) significantly demonstrated higher knowledge of ETS than non-trained nurses (P<0.005), while all other factors had no influence.

**Conclusions and recommendations:**

Majority of ICU nurses do not have desirable knowledge and skills of ETS, and are currently not following current ETS recommendations. This study has shown that training on ICU skills have positive influence to recommended ETS knowledge. We recommend ICU training, provision of clinical guidelines and adequate support to nurses employed in ICUs. Also, further studies using analytical approach to identify other factors beyond the scope of this study and testing the best approach in fostering adherence to ETS evidence-based recommendations are crucial.

## Introduction

Endotracheal suction (ETS) is a common invasive procedure which aims at keeping airways of the patient with endotracheal tube patent by mechanically removing accumulated pulmonary secretions[1–3]. Intubated patients often have an increased production of mucous and weakened ability to clear airway secretion which may pose some risks to the patient including infection (pneumonia) and atelectasis[4,5]. Therefore, ETS is a life-saving procedure to enhance clearance of respiratory tract secretion, improve oxygenation and prevent atelectasis [6].

Despite of ETS being a necessary procedure to both children and adult patients, if the procedure is not performed with correct techniques, it can lead to serious complications, such as bleeding, infection, hypoxia, bronchoconstriction, atelectasis, increase in intra-cranial pressure, cardiac arrest and sudden death [3,5,7–13]. Since the procedure can cause harm to the patient if it is done incorrectly, it is important therefore that Intensive Care Unit (ICU) nurses have the necessary knowledge and skills based on valid scientific evidence in performing ETS and aspects related to it. Wood (1998) as cited by Ansari et al (2012) demonstrated that, performing suctioning by well-educated nurses based on checking for patient's need, has better outcomes and fewer side effects than performing it routinely at every two hours [10]. One Cochrane review on the frequency of ETS to neonates identified only one article which was also outdated and therefore failed to recommend on the frequency of suctioning [14]. However, their review agrees on American Association for Respiratory Care Practice Guidelines that only when secretions are present, ETS suctioning can be performed, indicating that knowledge and skills of patient assessment is a pre-requisite.

As also indicated by many studies [5,7,15–18], a literature review by Pedersen et al (2009) isolated and recommended 11 guidelines for acceptable ETS that are associated with minimal complications. The recommendations are summarized in Table 1.

**Table 1:** Recommended guidelines for endotracheal suctioning.

SN	GUIDELINES	RECOMMENDED
1	Frequency	ETS should be performed only when necessary
2	Suctioning catheter	Should occlude less than half of the lumen of the ETT
3	Suctioning pressure	Should be lowest as much as possible, usually 80–120 mmHg
4	Depth of suctioning	Minimum invasion to the length of the ETT only
5	Time of suctioning	Should last no longer than 15 seconds
6	Continuous vs Intermitted	Should be continuous rather than intermittent suctioning during the individual suction procedure
7	Normal Saline instillation	No routine instillation of normal saline (N/S) prior to ETS
8	Oxygenation	There should be pre-oxygenation by the delivery of 100% oxygen for at least 30 seconds prior to and after the suctioning procedure to prevent decrease in oxygen saturation
9	Hyperinflation	Hyper-oxygenation prior to suctioning should be combined with hyperinflation (20–30 cmH_2_O)
10	Infection Control	Aseptic technique should be used for infection control
11	Closed vs Open suctioning	Both open and closed suction systems are recommended

Unsafe endotracheal suction practices remain the problem worldwide [2]. Although scientific evidence for the safe and efficient accomplishment of endotracheal suctions are available, many of these recommendations have not been observed in nurses' clinical practice, perhaps due to poor knowledge about this procedure [2,3]. Different evidence has shown that nurses often perform procedures traditionally / routinely, regardless of established ETS evidence-based recommendations. That indicates the existence of gaps between scientific knowledge and common practice [19–22], and the reasons identified for nurses not to incorporate the recommendations into clinical practice include: resistance to change, little support from managers, lack of ICU training, lack of ease access to the literature, and lack of time to read and understand the literature, competing workload pressures, poor change management process and lack of access to guidelines [19,23,24].

In Tanzania, the knowledge and practice of ETS for intubated patients using the available evidence-based recommendations are under-researched and therefore neither the prevailing knowledge and practice nor the induced complications from ETS practice of ICU nurses are well known. The study done by Said (2012) at Muhimbili National Hospital on knowledge and practice on prevention of ventilator associated pneumonia among ICU nurses reported only one item of ETS recommendation (practice of hand washing before and after ETS procedure), and only involved a small sample of nurses [25].

We therefore report the knowledge and practice of ICUs nurses on ETS among ICU intubated patients in five hospitals of Dar es Salaam, Tanzania.

## Methods

### Design and settings

This study used a descriptive cross-sectional observational design. Five hospitals with ICUs in Dar es Salaam namely Muhimbili National Hospital (MNH), Muhimbili Orthopaedic Institute, Agakhan hospital, Hurbert Kairuki Memorial Hospital, and T. M. J Hospital were involved. These were the only hospitals in Dar es Salaam with ICUs where the population of nurses who practice ETS could be found.

### Study population

The study population involved nurses working in ICUs of five hospitals in Dar es Salaam. They comprise of 122 nurses, and their ICUs provide care to both children and adults. Due to their small population, all were targeted to respond to the questionnaire and be assessed for endotracheal tube suctioning practices. A total of 19 participants were excluded from the study (6 nurses were on annual leave, 4 on study leave, 5 nurses were the ICU managers, not providing bedside care and 4 refused to consent for the study). The remaining 103 (84.4%) signed the informed consent and took part in the study. Of 103 nurses who consented for the study, all of them responded to the questions and 35 nurses were observed for their endotracheal suction practices.

### Data collection tools

Self-administered questionnaire and observational checklist were used to collect data. Both questionnaire and observational checklist were developed by the research group by adapting the constructs from the current ETS recommendations [26]. The tools were then reviewed by the colleagues who were not part of the investigation team, and then pretested to ten (10) nurses working in the Highly Dependent Unit (HDU) of the Muhimbili National Hospital, where also ETS was routinely done as one of the procedures to patients in the unit. Through pretesting of the tools, one question in the questionnaire was corrected for clarity. The questionnaire consisted of 6 items on demographic information, and 19 structured closed ended multiple-choice questions assessing knowledge on ETS in relation to evidence-based recommendations.

The observation checklist was adopted from Jansson et al 2013 with few items removed or slightly altered in reflection of the local practice of Tanzanian context [2].

### Data collection procedures

#### Administering the questionnaire

Participants were recruited by research assistants under the supervision of the first Author (E.T.M). The research assistants were nurses working in different departments within the participating hospitals. To each participant, detailed information about this research was provided on first encounter and then each participant was asked to sign a consent form. After acceptance, every participant was given the questionnaire to self-read the questions and then provide responses. Participants were given at least two hours to complete the questionnaire before collecting it back. All participants were able to read the questions and provide answers without needing clarifications from the research assistants.

#### Observation of practice

Observation of practice included the overall procedure of endotracheal tube suctioning of the intubated patients. This was done through observation of nurse- patient interaction during suction procedure using standardized checklist and measured against evidence-based recommendations. Stopwatch was used in determining the duration of suctioning, and the optimal cuff pressure by observing nurses inflating the cuff slowly until the wheezing sound disappear or deflating to loosen the tight cuff. Observation was conducted on 34% (n = 35) nurses in a natural ICU environment. Nurses from all the five hospitals contributed to observation data: Muhimbili National Hospital, n = 10, Muhimbili Orthopedic Institute, n = 8, Aga-khan Hospital, n = 6, Hurbert Kairuki Memorial Hospital, n = 5, and T. M. J Hospital, n = 6. Nurses were made aware during informed consent that they were going to be observed for their practice however they were not given the details of items being observed. Two observation encounters were made and the average score for the two observations for each nurse was considered final. All observation data were collected by the first author. Four items were assessed during observation i.e.

- **Practices prior to ETS event–**patient assessment, chest auscultation, pre-suctioning hyper-oxygenation, and cuff pressure checking.
- **Infection-control practice–**hand disinfection prior to suctioning, self-protection, and maintaining sterility of suction catheter until inserted into airway.
- **The ETS events–**sodium chloride instillation, size of suction catheter (≤ half of internal diameter of ETT), number of suction passes ≤ 2, duration of suction applied to airway (< 15 seconds), level of suction pressure 80-150mmHg
- **Post ETS practice–**patient reconnected to oxygen within 10 seconds post suctioning, post-suctioning hyper oxygenation, post-ETS assessments: patients’ chest auscultation after suctioning, patient reassured, hand disinfection post suctioning, proper way of disposing used catheter and gloves post suctioning, cuff pressure checked.

### Data analysis

Data from both structured questionnaires and checklist were analyzed using SPSS program version 20. Demographic data categorized into gender, level of education, years of working in ICU, and ICU training were analyzed for descriptive statistics, which in turn were used as independent variables for their relationship with knowledge and practice of ETS. Scoring of knowledge and practice were made in percentile based on the number of items a participant would score. Each questionnaire and checklist items were valued at 1-point score, then the average of score for both knowledge and practice was calculated in percentages. The quality of performance was categorized into three levels based on the sum of scores to individual items; desirable (76–100%), moderate (50–75%) and undesirable (< 50%) [10]. Chi-square, Fisher’s exact test and mid P exact test were used to identify the significance in the observed differences in knowledge and practice of ETS scores by the independent variables: level of education, years of working in ICU, and ICU training, considering statistically significant at p-value less than 0.05. All results assumed a two-tailed distribution with an alpha level of 5%.

### Ethical consideration

Ethical approval was given by the Muhimbili University of Health and Allied Sciences Institutional Review Board (Reference No. MU/PGS/SAEC/Vol.XI/234) and permission granted by the respective hospitals. All participants were given detailed information about the study and then signed a consent form before data collection began. During consent process, they were given both verbal and written information about the voluntary nature of participation, the guarantee of confidentiality of their information, and the freedom they had to withdraw from the study any time if they felt so; without any consequences to their rights as workers. Numbers were used in the questionnaires instead of names to ensure confidentiality.

## Results

One hundred three (103) ICU nurses participated in the study, response rate 96% of potential study participants. The majority (60.2%) of study participants had diploma in Nursing. More than half (57.3%) had intensive care training, 43.7% had worked in ICU between 1 and 5 years and 72.7% were females “**Table 2”**

**Table 2:** Participants demographic characteristics.

Factors	Variables	Frequency	Percent
Nursing Education (n = 103)	Certificate	18	17.5
	Diploma	62	60.2
	Degree	23	22.3
	Master with specialization	-	-
ICU Training (n = 103)	Yes	59	57.3
	No	44	42.7
Work Experience (n = 103)	< 1 year	31	30.1
	1–5 years	45	43.7
	6–10 years	19	18.4
	> 10 years	8	7.8
Gender (n = 103)	Male	28	27.2
	Female	75	72.8

### Participants’ level of knowledge of endotracheal suctioning

Majority of ICU nurses (69.9%) knew the indication of ETS, and 77.7% knew the action to be taken in case of abrupt changes in Electrocardiogram (ECG) monitor but not doing the procedure as per guidelines. However, most (86.4%) didn’t know how to choose the appropriate catheter size for the child, and 88.3% didn’t know the appropriate way of suctioning the child **“Table 3”**.

**Table 3:** Overall scores for level of knowledge.

Level	Number of respondents (n = 103)	Percent (%)
**76–100%**	-	-
**50–75%**	20	19.4
**< 50%**	83	80.6

### Factors associated with knowledge

There was no significant diference in knowledge between nurses with different levels of nursing education (p = 0.610), and ICU experiences (p = 0.161). However, nurses who had received ICU training demonstrated significantly higher knowledge than those who did not receive training (*X*^2^ = 3.854, p = 0.049). **Table 4** summarizes the findings of factors associated with knowledge.

**Table 4:** Factors associated with knowledge.

Factors	Variables	No. of respondents(%) n = 103	No. of moderate score within the category(%)	*X*^2^ or Fishers exact test of the observed difference	P value
Level of Nursing education	Certificate	18(17.5%)	2(11.1%)	Fisher’s exact test	0.610
	Diploma	62(60.2%)	14(22.6%)		
	Degree	23(22.3%)	4(17.4%)		
ICU training	Yes	59(57.3%)	16(27.1%)	3.854	0.049*
	No	44(43.7%)	5(11.6%)		
Year of experience	< 1year	31(30.1%)	4(12.9%)	Fisher’s exact test	0.161
	1–5 years	45(43.7%)	7(15.6%)		
	6–10 years	19(18.4%)	7(36.8%)		
	>10 years	8(7.8%)	2(25.0%)		

X^2^, Chi square

*, statistically significant.

### Adherence to ETS evidence based recommendations during practice

Overall, the majority (n = 30, 85.7%) of the observed participants scored less than the desired level (< 50% score), only 5 participants (14.3%) scored at the moderate level (50–75% score) and none scored at the desirable level (76–100% score).

Taking moderate score as the score criteria, there was no association between practice of endotracheal tube suctioning in relation to recommended guidelines and level of education and ICU training. There was no significant difference in practice between those with ICU training and those without (p = 0.812), across the three levels of nursing education (p = 0.356) and across the three strata of nurses’ work experiences (p = 0.791). Findings for level of practice in relation to level of nursing education, ICU training and work experience are summarized in “**Table 5”.**

**Table 5:** Factors associated with practices.

Factors	Variables	No. of respondents observed (%) n = 35	No. of respondents scored moderate	*X*^2^ or fishers exact test of the observed difference	P value
Level of nursing education	Certificate	4(11.4%)	1	Fisher’s exact	0.356
	Diploma	17(48.6%)	4		
	Degree	14(40.0%)	-		
ICU training	Yes	19(54.3%)	3	Mid P exact test	0.812
	No	16(45.7%)	2		
Work experience	< 1 year	12(34.3%)	1	Fisher’s exact test	0.791
	1 – 5years	13(37.1%)	3		
	6 – 10years	6(17.1%)	-		
	>10 years	4(11.4%)	1		

### Practice in relation to recommended guidelines

Technical discrepancies were observed in practices prior and post ETS events. All nurses observed did not auscultate chest 35(100%), did not pre-oxygenate the patient 30(85.7%), and did not check cuff pressure 35(100%) prior to suctioning. Likewise, at post suctioning 26 (74.3%) did not auscultate chest, 27(77.1%) did not hyper-oxygenate the patient and 33(94.3%) did not check cuff pressure. Furthermore, a large proportion of participants were observed not reassuring the patients after the procedure 33(94.3%) whereas 27(77.1%) did not disinfect their hands post-suctioning procedure. Infection control practices were observed to be high on self-protection 34(97.1%), and maintenance of catheter sterility until inserted into the airway 33(94.3%). **Table 6** provides the summary of observed practices of ETS in relation to current recommendations.

**Table 6:** ICU nurses’ practice in relation to current recommendations prior to, during and post ETS events.

Practices prior to, during and post ETS event (n = 35)	Recommended guidelines for ETS	No.(%) Observed	No.(%) Not observed
Practices prior to ETS event	Patient assessment: Patient’s chest auscultation before ETS	-	35(100%)
	Pre-suctioning hyper-oxygenation	5(14.3%)	30(85.7%)
	Cuff pressure checked	-	35(100%)
	Hand disinfection prior to suctioning	15(42.9%)	20(57.1%)
	Self-protection observed	34(97.1%)	1(2.9%)
	Sterility of suction catheter maintained until inserted into airway	33(94.3%)	2(5.7%)
The ETS event	Sodium chloride instillation	19(54.3%)	16(45.7%)
	Size of suction catheter (≤ Half of internal diameter of ETT)	20(57.1%)	15(42.9%)
	Number of suction passes ≤ 2	17(48.6%)	18(51.4%)
	Duration of suction applied to airway (< 15 seconds)	14(40.0%)	21(60.0%)
	Level of suction pressure 80-120mmHg	21(60.0%)	14(40.0%)
Post-ETS practices	Patient reconnected to oxygen within 10 seconds post suctioning	17(48.6%)	18(51.4%)
	Post-suctioning hyper oxygenation	8(22.9%)	27(77.1%)
	Post-ETS assessments: Patient’s chest auscultation after suctioning	9(25.7%)	26(74.3%)
	Patient reassured	2(5.7%)	33(94.3%)
	Hand disinfection post suctioning	8(22.9%)	27(77.1%)
	Proper way of disposing used catheter and gloves post suctioning	18(51.4%)	17(48.6%)
	Cuff pressure checked	2(5.7%)	33(94.3%)

## Discussion

Studies about knowledge and practice of ETS among nurses have been conducted in many countries as they predict the safety of patients in critical conditions [6,10,23]. However, little is known from the Tanzanian settings. The fact that about 85% of all nurses who practice at the studied ICUs in Dar es Salaam city were reached and included in the study, with careful management of data and focused analysis, the authors think that the findings are valuable in providing a good picture of safety situation of the critical patients in Dar es Salaam. The findings however, cannot be generalized to the whole Tanzania for the fact that this study did not involve nurses in other referral hospitals outside Dar es Salaam (Kilimanjaro Christian Medical Centre, Bugando Medical Centre and Mbeya Zonal Referral Hospital) which also have ICUs and may have different ICU practice protocols.

Findings of this study enlighten that majority 62(60.2%) of the nurses practicing in the ICUs in Dar es Salaam are diploma holders in nursing education. The high percentage of diploma holders could be explained by the fact that the public service system has only recently started recruitment of nurses at bachelors and masters level, believing that these cadres could have better knowledge and skills of caring for critical patients in the ICUs. The curricula for nurses’ training at certificate and diploma levels in Tanzania does not cover the care of critically ill patients, where ETS is part of the package; thus, nurses learn through observation from experienced ones, or through informal training in or outside the hospital.

Although, more than a half 59(57.3%) of respondents had undergone ICU training, the findings however did not reflect the relationship of their training and the percentage of overall scores in knowledge of the ETS recommendations. This is spotted with higher scores (80.6%) of undesired level of knowledge of ETS recommendations compared to those who scored at the moderate level (19.4%) and the desired level (0%). This finding is similar to that of Day et al (2001) who also reported low level of knowledge among many ICU nurses in London–United Kingdom [27]. The findings are however, contrasted by the findings of Ansari et al (2012) where higher levels of knowledge among nurses than performance was observed [10]. The present study findings may imply that the trainings given were not sufficient, not as per performance indicators or were not focused in addressing the required skills by ICU nurses [28–30].

Even though it is widely known that infection-control practices are crucial elements in the prevention of cross-infections and transmission of pathogens through hands or equipment [31], this study found that only about half of the nurses would disinfect their hands before ETS procedure and only 22.9 percent disinfected their hands after the procedure. This practice among ICU nurses in Dar es Salaam ICUs alerts a practice that may increase risk of acquiring nosocomial infection.

ETS evidence based recommendations suggests not to administer N/S routinely before suctioning [1], since it can cause oxygen desaturation [32]. However, it was observed in more than half of the respondents in this study, contradicting the ETS recommendations. Nevertheless, as researchers in the less resource settings, we feel that the benefit of saline instillation to patients under non-humidified ventilation may outweigh the disadvantages even though its practice may need caution. While there are few current systematic reviews and meta-analysis [33–35] indicating reduced oxygen 5 minutes post ETS with estimated mean difference of −1.14 (95%CI, −2.25 to −0.03) [34], while also admitting the poor quality of included studies, we perceive that these findings are outweighed by the previous review by Paratz and Stockton of 2009 who concluded that N/S instillation may not be harmful in terms of hemodynamics, gas exchange, increased dyspnea or distress [36]. Studies recommend ventilation with humidified oxygen [37], however, this study was conducted in the settings where patients are commonly ventilated with non-humidified air due to lack of humidifiers, which is expected to lead to drying the mucous membranes of the airways. Suction of the secretions after ventilation with non-humidified air without instillation with normal saline may damage this dried mucous membrane, leading to bleeding and reactions with the ultimate impact to ventilator associated pneumonia. Moreover, studies that disprove instillation of N/S fail to provide solutions of what could be the effective way of ETS where patients had been ventilated with non-humidified air such as in low resource settings.

Also, the study found that although majority of the respondents instilled N/S prior to ETS, most of them did not know the function of N/S to ETS procedure, did not know when to instill and the amount of N/S to be used in adult and children. It is not known whether the practice of instilling NS is a set protocol by the institutions or not since the authors did not find SOPs for ETS placed in the ICUs. Previous studies show that correct performance by nurses in endotracheal suctioning can minimize its undesirable side effects to the patients [27,38,39]. Nurses can, by utilizing evidence-based recommendations in their practice, improve the quality of care hence prevention of unnecessary deaths as well as medical interventions, and decrease the period of hospitalization and patients' expenses.

Our study found the gap between knowledge and practice as pointed out in other studies [10,11,19,27,39]. While nurses who had ICU training demonstrated good knowledge than those who did not have training, this difference was not apparent in their practice. Ansari’s research group argues that, theoretical education which is often given as part of continuing courses for nurses may not be adequate to influence change in practical skills needed by the critical care nurses; the situation which our authors think it might have been the case for ICU nurses who had received training in this study, [10]. On the other hand, some of the findings in this study showing failure to adhere to ETS evidence-based recommendations, especially on chest auscultation, hyper oxygenation, hand disinfection and cuff pressure checkup prior to, and post endotracheal suctioning events are controversial in conclusion. As it was apparent that nurses who received ICU training demonstrated knowledge of these recommendations, however could not demonstrate their practice, raises curiosity as to whether the observed poor practice was related to lack of knowledge and skills or carelessness [40]. These phenomena, however; call for further studies to determine the predictors of knowledge and practice gap in our settings.

The promising circumstance is that some studies have revealed that low level of knowledge and practice of nurses can be improved if training that reflect needs is conducted according to established protocol [11,19,39,41].

## Strengths and limitations

The instrument used for assessing nurses’ knowledge on ETS was based on current ETS recommendations and reviewed by other colleagues which justifies its validity. However, lack of psychometric testing may limit the comparability of our findings with others. Also, in the analysis of practice, it was not possible to eliminate the Hawthorne effect in the sense that the presence of the researcher somehow affected nurses' practice. However, to minimize this effect, the researcher established a good relationship with nurses and worked in the respective ICUs for three days for nurses to get used with her presence and feel relaxed during their practice, before data collection began [42]

## Conclusions and recommendations

Despite of the possible Hawthorne effect and lack of psychometrics for the assessment instrument, this study found that practice of ICU nurses was not in line with current ETS recommendations. We observed significant discrepancies in knowledge and practices in relation to current recommendations as well as contrasting relationship between knowledge and its corresponding practice. The perceived barriers could be resistance to change, insufficient training, and lack of support from higher authority. The fact that unsafe ETS practices may jeopardize patient safety, and thus the quality of nursing care, we recommend innovative educational interventions on evidence based ETS recommendations, clinical guidelines coupled with adequate support like mentorship, support systems like guidelines and SOPs be in place. Learning resources such as articles, journals and electronic resources such as computers and internet would perhaps increase nurses’ behavior to use evidence-based information in enhancing their skills in their practice. Further studies using analytical approach to identify other factors beyond the scope of this study and testing the best approach in fostering adherence to ETS evidence-based recommendations may be needed.

## Supporting information

S1 File Data for sharing. (XLSX)

S2 File Questionnaires. (DOCX)

## References

[1] Spears N, Cook N, Garcia K. Instillation of Normal Saline in Endotracheal Suctioning. Pharm Nurs Student Res Evidence-Based Med Poster Sess Pap 15 [Internet]. 2012 [cited 2015 Jan 4]; Available from: http://digitalcommons.cedarville.edu/pharmacy_nursing_poster_session/15/

[2] Jansson M, Ala-Kokko T, Ylipalosaari P, Kyngäs H. Evaluation of endotracheal-suctioning practices of critical-care nurses–An observational correlation study. J Nurs Educ Pract [Internet]. 2013 1 14 [cited 2014 Dec 17];3(7):99–105. Available from: http://www.sciedu.ca/journal/index.php/jnep/article/view/1586

[3] Favretto DO, Silveira RC, Canini SR, Garbin LM, Martins FT DM. Endotracheal Suction in Intubated Critically Ill Adult Patients Undergoing Mechanical Ventilation: a Systematic Rewiew. Rev Lat Am Enferm. 2012;20(5):997–1007. 10.1590/s0104-11692012000500023 23174846

[4] Morrow, Brenda M. PhD; Argent ACFcpS. A comprehensive review of pediatric endotracheal suctioning: Effects, indications, and clinical practice*. BJU International. 2008 p. 465–77. 10.1097/PCC.0b013e31818499cc 18679146

[5] Royal University Hospital, Saskatoon City Hospital, St. Paul’s Hospital. Suctioning–Pediatric / Neonate Patients Ventilated (Conventional and High Frequency) Via Artificial Airways. 2017.

[6] Haghighat S, Yazdannik A. The practice of intensive care nurses using the closed suctioning system: An observational study. Iran J Nurs Midwifery Res [Internet]. 2015;20(5):619–25. Available from: http://www.ncbi.nlm.nih.gov/pubmed/26457102%5Cnhttp://www.pubmedcentral.nih.gov/articlerender.fcgi?artid=PMC4598911 10.4103/1735-9066.164509 26457102 PMC4598911

[7] Pedersen C, … MR-N. La aspiración endotraqueal del paciente adulto intubado—¿Cuál es la evidencia? Intensiva, Crítica … 2009.

[8] Day T, Farnell S, Haynes S, Wainwright S, Wilson-Barnett J. Tracheal suctioning: an exploration of nurses’ knowledge and competence in acute and high dependency ward areas. J Adv Nurs [Internet]. 2002 Jul [cited 2015 Jan 4];39(1):35–45. Available from: http://doi.wiley.com/10.1046/j.1365-2648.2002.02240.x 12074750 10.1046/j.1365-2648.2002.02240.x

[9] Davies K. Determining Standard Criteria for Endotracheal Suctioning in the Paediatric Intensive Care Patient: An Exploratory Study. Determining Standard Criteria for Endotracheal Suctioning in the Paediatric Intensive Care Patient: An Exploratory Study. 2008;(September):157–76. 10.1016/j.iccn.2011.01.002 21371887

[10] Ansari A, Alavi NM. The gap between knowledge and practice in standard endo-tracheal suctioning of ICU nurses, Shahid Beheshti Hospital. … Crit Care Nurs. 2012;5(2):71–6.

[11] Hadian ZS, Sabet RS. The effect of endotracheal tube suctioning education of nurses on decreasing pain in premature neonates. Iran J Pediatr. 2013;23(3):340–4. 23795259 PMC3684481

[12] Özden D, Görgülü RS. Development of standard practice guidelines for open and closed system suctioning. J Clin Nurs [Internet]. 2012 5 [cited 2015 Jan 4];21(9–10):1327–38. Available from: http://www.ncbi.nlm.nih.gov/pubmed/22390197 10.1111/j.1365-2702.2011.03997.x 22390197

[13] Fisk AC. The effects of endotracheal suctioning in the pediatric population: An integrative review. Dimens Crit Care Nurs. 2018;37(1):44–56. 10.1097/DCC.0000000000000275 29194174

[14] Bruschettini M, Zappettini S, Moja L, Calevo MG. Frequency of endotracheal suctioning for the prevention of respiratory morbidity in ventilated newborns. Cochrane Database Syst Rev. 2015;2015(1). 10.1002/14651858.CD011493.pub2 PMC8915721 26945780

[15] Suctioning E, Ventilated M, With P, Airways A. AARC Clinical Practice Guidelines Endotracheal Suctioning of Mechanically Ventilated Patients With Artificial Airways 2010. 2010;(January 1990):758–64. 20507660

[16] Rolls K, Rand Butcher, Smith K, Kent B, Jones P, Chaboyer W, et al. Suctioning an Adult with a Tracheal Tube NSW Health Statewide Guidelines for Intensive Care. Queensland, Australia; 2010.

[17] Martin KT. Suctioning the Airway Vol. 5798 Riverside, CA: RC Educational Consulting Services, Inc.; 2008.

[18] Morrow B, Futter M, Argent A. Effect of endotracheal suction on lung dynamics in mechanically-ventilated paediatric patients. Aust J Physiother [Internet]. 2006 [cited 2015 Jan 4];52. Available from: http://www.sciencedirect.com/science/article/pii/S0004951406700472 10.1016/s0004-9514(06)70047-2 16764549

[19] Ania González N, Martínez Mingo A, Eseberri Sagardoy M, Angeles Margall Coscojuela M., Carmen Asiain Erro M. Evaluación de la competencia práctica y de los conocimientos científicos de enfermeras de UCI en la aspiración endotraqueal de secreciones. Enfermería Intensiva [Internet]. 2004 1 [cited 2015 Jan 4];15(3):101–11. Available from: http://linkinghub.elsevier.com/retrieve/pii/S1130239904781511 15450150 10.1016/s1130-2399(04)78151-1

[20] Swartz K, Noonan DM, Edwards-Beckett J. A national survey of endotracheal suctioning techniques in the pediatric population. Hear Lung J Acute Crit Care [Internet]. 2017 10 5;25(1):52–60. Available from: 10.1016/S0147-9563(96)80013-6 8775871

[21] Negro A, Ranzani R, Villa M, Manara D. Survey of Italian intensive care unit nurses’ knowledge about endotracheal suctioning guidelines. Intensive Crit Care Nurs [Internet]. 2014;30(6):339–45. Available from: 10.1016/j.iccn.2014.06.003 25193542

[22] Leddy R, Wilkinson JM. Endotracheal suctioning practices of nurses and respiratory therapists: How well do they align with clinical practice guidelines? Can J Respir Ther CJRT = Rev Can la Ther Respir RCTR [Internet]. 2015;51(3):60–4. Available from: http://www.ncbi.nlm.nih.gov/pubmed/26283870%5Cnhttp://www.pubmedcentral.nih.gov/articlerender.fcgi?artid = PMC4530836 PMC4530836 26283870

[23] Leddy R, Wilkinson JM. Endotracheal suctioning practices of nurses and respiratory therapists: How well do they align with clinical practice guidelines? Can J Respir Ther CJRT = Rev Can la Ther Respir RCTR. 2015;51(3):60–4. PMC4530836 26283870

[24] Negro A, Ranzani R, Villa M, Manara D. Survey of Italian intensive care unit nurses’ knowledge about endotracheal suctioning guidelines. Intensive Crit Care Nurs. 2014;30(6):339–45. 10.1016/j.iccn.2014.06.003 25193542

[25] Said AT. Knowledge and practice of intensive care nurses on prevention of Ventilator Associated Pneumonia At Muhimbili National Hospital, Dar ES Salaam Tanzania [Internet] MSc Nursing (Critical Care and Trauma) Dissertation. Muhimbili University of Health and Allied Sciences; 2012 Available from: http://ir.muhas.ac.tz:8080/jspui/bitstream/123456789/588/1/2012-11-05-CORRECTEDyamwisho.pdf

[26] Pedersen C, M R-N, J H, I E. Endotracheal suctioning of the adult intubated patient—What is the evidence? IIntensive Crit Care Nurs 2009 [Internet]. 2009 [cited 2015 Jan 4];25(1):2009 Available from: http://www.sciencedirect.com/science/article/pii/S0964339708000566 10.1016/j.iccn.2008.05.004 18632271

[27] Day T, Wainwright SP, Wilson-Barnett J. An evaluation of a teaching intervention to improve the practice of endotracheal suctioning in intensive care units. J Clin Nurs [Internet]. 2001 9 15 [cited 2015 Jan 4];10(5):682–96. Available from: http://doi.wiley.com/10.1046/j.1365-2702.2001.00519.x 11822519 10.1046/j.1365-2702.2001.00519.x

[28] Quinton S, Higgins Y. Critical care training needs analysis for ward staff. Crit Care [Internet]. 2003 3 3;7(Suppl 2):P239–P239. Available from: http://www.ncbi.nlm.nih.gov/pmc/articles/PMC3301684/

[29] Kol E, İlaslan E, Turkay M. Training needs of clinical nurses at an university hospital in Turkey. Nurse Educ Pract. 2017;22:15–20. 10.1016/j.nepr.2016.11.004 27889623

[30] Haniffa R, Lubell Y, Cooper BS, Mohanty S, Alam S, Karki A, et al Impact of a structured ICU training programme in resource-limited settings in Asia. PLoS One. 2017;12(3):1–13. 10.1371/journal.pone.0173483 PMC5349661 28291809

[31] Pittet D, Allegranzi B, World Health Organization World Alliance for Patient Safety Challenge Core Grroup of Experts. The World Health Organization guidelines on hand hygiene in health care and their consensus recommendations. Infect Control Hosp Epidemiol [Internet]. 2009 [cited 2015 Jan 4];30(7):600379 Available from: http://www.jstor.org/stable/10.1086/600379 10.1086/600379 19508124

[32] Iranmanesh S, Rafiei H. Normal saline instillation with suctioning and its effect on oxygen saturation, heart rate, and cardiac rhythm. Int J Nurs Educ [Internet]. 2010 [cited 2015 Jan 4];1(2). Available from: http://www.i-scholar.in/index.php/ijne/article/view/46123

[33] Caparros ACS, Forbes A. Mechanical ventilation and the role of saline instillation in suctioning adult intensive care unit patients: An evidence-based practice review. Dimens Crit Care Nurs. 2014;33(4):246–53. 10.1097/DCC.0000000000000049 24895955

[34] Wang CH, Tsai JC, Chen SF, Su CL, Chen L, Lin CC, et al Normal saline instillation before suctioning: A meta-analysis of randomized controlled trials. Aust Crit Care [Internet]. 2017;30(5):260–5. Available from: 10.1016/j.aucc.2016.11.001 27876258

[35] Ayhan H, Tastan S, Iyigun E, Akamca Y, Arikan E, Sevim Z. Normal saline instillation before endotracheal suctioning: “What does the evidence say? What do the nurses think?”: Multimethod study. J Crit Care [Internet]. 2015;30(4):762–7. Available from: 10.1016/j.jcrc.2015.02.019 25841280

[36] Paratz J, Stockton K. Efficacy and safety of normal saline instillation: a systematic review. Physiotherapy [Internet]. 2009 [cited 2015 Jan 4];95(4):241–50. Available from: http://www.sciencedirect.com/science/article/pii/S0031940609000625 10.1016/j.physio.2009.06.002 19892088

[37] Al Ashry HS, Modrykamien AM. Humidification during mechanical ventilation in the adult patient. Biomed Res Int. 2014;2014. 10.1155/2014/715434 PMC4096064 25089275

[38] Maggiore SM, Lellouche F, Pignataro C, Girou E, Maitre B, Richard J-CM, et al Decreasing the Adverse Effects of Endotracheal Suctioning During Mechanical Ventilation by Changing Practice. Respir Care. 2013;58(10):1588–97. 10.4187/respcare.02265 23466423

[39] Kelleher S, Andrews T. An observational study on the open-system endotracheal suctioning practices of critical care nurses. J Clin Nurs [Internet]. 2008 2 [cited 2015 Jan 4];17(3):360–9. Available from: http://www.ncbi.nlm.nih.gov/pubmed/18205692 10.1111/j.1365-2702.2007.01990.x 18205692

[40] Reader TW, Gillespie A. Patient neglect in healthcare institutions: a systematic review and conceptual model. BMC Health Serv Res [Internet]. 2013;13(1):156 Available from: http://bmchealthservres.biomedcentral.com/articles/10.1186/1472-6963-13-156 23631468 10.1186/1472-6963-13-156 PMC3660245

[41] Kargar M, Shirazi ZH, Edraki M, Pishva N. The Effects of ETT Suction Education on the Knowledge and Performance of Intensive Care Nurses. Anaesthesia, Pain Intensive Care. 2008;12(1):5–10.

[42] Oswald D. Handling the Hawthorne effect: The challenges surrounding a participant observer. Rev Soc Stud [Internet]. 2014;1(1):53–73. Available from: http://www.rossjournal.co.uk/wp-content/uploads/2016/12/RoSS-Vol1-No1-Oswald-et-al-53-73.pdf

