# Supplementary material for: Intensive care nurses’ knowledge and practice on endotracheal suctioning of the intubated patient: A quantitative cross-sectional observational study
Source: PLoS One. 2018 Aug 16;13(8):e0201743. doi: 10.1371/journal.pone.0201743 (PMC6095500; doi:10.1371/journal.pone.0201743)
Supplement: S2 File — (DOCX) [file pone.0201743.s002.docx]

**Supporting information, I: Questionnaires**

Instructions:

- Answer all questions
- Select only one correct answer and fill in the provided box.
- Do not write your name, use the given number to ensure confidentiality.

**Part One**: Identification

1. Date: ………………………

2. Number of interviewee………………

3. Level of nursing education:

1. Certificate
2. Diploma
3. Degree
4. Master with specialization in……………………………………………

4. Undergone ICU Training:

1. Yes
2. No

5. Working experience in the unit:

1. < 1 year
2. 1 – 5 years
3. 6 – 10 years
4. > 10 years

6. Gender:

1. Male
2. Female

**Part Two**: Interview

**Knowledge Assessment**

1. What is the primary indication for endotracheal suctioning?

1. Presence of pneumonia
2. Presence of atelectasis
3. Ineffective coughing
4. Retention of secretions

2. Complications of endotracheal suctioning include all of the following except:

1. Bronchospasm
2. Hyperinflation
3. Mucosal trauma
4. Elevated intracranial pressure

3. How often should patients be suctioned?

1. At least once every 2 to 3 hours
2. More than once every 2 to 3 hours
3. Once every 6 hours
4. Only when necessary

4. What is the normal range of negative pressure to use when suctioning an adult patient?

1. –80 to –120 mm Hg
2. –80 to –100 mm Hg
3. –60 to –80 mm Hg
4. –20 to –30 mm Hg

5. What is the normal range of negative pressure to use when suctioning children?

1. –60 to –80 mm Hg
2. –80 to –100 mm Hg
3. –100 to –120 mm Hg
4. –150 to –200 mm Hg

6. You are about to suction a 10-year-old patient who has a 6-mm (internal diameter) endotracheal tube in place. What is the maximum size of catheter that you would use in this case?

1. 6Fr
2. 8Fr
3. 10Fr
4. 14Fr

|  |
| --- |

7. You are about to suction a female patient who has an 8-mm (internal diameter) endotracheal tube in place. What is the maximum size of catheter you would use in this case?

1. 8Fr
2. 10Fr
3. 12Fr
4. 14Fr

8. At what depth the suctioning catheter should be inserted?

1. At half of ETT
2. At quarter to ETT
3. To the length of ETT
4. To the length beyond ETT

9. To prevent hypoxemia when suctioning a patient, the respiratory care practitioner should initially do which of the following?

1. Manually ventilate the patient with a resuscitator.
2. Pre-oxygenate the patient with 100% oxygen.
3. Give the patient a bronchodilator treatment.
4. Have the patient hyperventilate for 2 minutes

10. To maintain positive end-expiratory pressure (PEEP) and high FIO2 when suctioning a mechanically ventilated patient, what would you recommend?

1. Limit suction time to no more than 5 seconds.
2. Use a closed-system multiuse suction catheter.
3. Limit suctioning to once an hour.
4. Use the smallest possible catheter.

11. Total application time for endotracheal suction in adults should not exceed which of the following?

1. 20 to 25 seconds
2. 15 to 20 seconds
3. 10 to 15 seconds
4. 3 to 5 seconds.

12. Which is the appropriate way of endotracheal suctioning?

a) Suctioning alternatively continuously and intermittently

b) Suctioning 1^st^ continuously followed by intermittently

c) Suctioning continuously

d) Suctioning intermittently

13. While suctioning a patient, you observe an abrupt change in the electrocardiogram wave form being displayed on the cardiac monitor. Which of the following actions would be most appropriate?

1. Change to a small catheter and repeat the procedure
2. Stop suctioning and immediately administer oxygen
3. Stop suctioning and report your finding to the in-charge/supervisor
4. Decrease the amount of negative pressure being used.

14. Which of the following methods can help to reduce the likelihood of atelectasis due to endotracheal suctioning?

1. Limit the amount of negative pressure used.

2. Hyper-inflate the patient before and after the procedure.

3. Suction for as short a period of time as possible.

a) 1 and 2

b) 1 and 3

c) 2 and 3

d) 1, 2, and 3

15. Which of the following can help to minimize the likelihood of mucosal trauma during suctioning?

1. Use as large a catheter as possible a) 1 and 2
2. Rotate the catheter while withdrawing. b) 2 and 4
3. Use as rigid a catheter as possible. c) 2 and 3
4. Limit the amount of suctioning pressure d) 1, 2 and 4

16. The instillation of sterile 0.9% saline should not be done on a routine basis but may be required for tenacious secretions. Which of the following is a function of Normal saline?

1. Prevent thick secretions
2. Stimulates a cough that moves secretions.
3. Provides a lavage that moves secretion
4. Both b and c

17. If required, instill sterile 0.9% saline into the tube. At what episode the sterile N/S 0.9% should be instilled?

1. During inspiration
2. During expiration
3. During quite period
4. a and b are correct

18. What is the normal amount of normal saline to be instilled to adult patients?

1. 0. 3 – 1ml
2. 0.5 – 1.5mls
3. 1.5 – 2mls
4. 2 – 3 mls

19. What is the normal amount of normal saline to be instilled to an infant patient?

1. 0. 3 – 1ml
2. 0.5 – 1.5mls
3. 1.5 – 2mls
4. 2 – 3 mls
